# The Human Ear

**Published:** 1892-09

**Authors:** 


### THE HUMAN EAR.

Few people realize what a wonderfully delicate piece of mechanism the human ear really is. That which we ordinarily designate as the "ear," is, after all only the mere outer porch of a series of winding passages which lead from the world without to the world within. Certain of these passages are filled with liquid, besides having membranes stretched like parchment curtains across the corridor at different points. When a sound wave strikes these they are thrown into vibrations and made to tremble like the head of a drum does when struck with a stick or with the fingers. Between two of these parchment-like curtains a chain of minute bones extend, which serve to tighten or relax the membranes and to communicate vibrations to them. In the innermost place of all, a row of white threads, called nerves, stretch like the strings of a piano from the last point from which the tremblings reach, passing thence inward to the brain.

